# Weather Sensing in an Urban Environment with the Use of a UAV and WebRTC-Based Platform: A Pilot Study

**DOI:** 10.3390/s21217113

**Published:** 2021-10-26

**Authors:** Agnieszka Chodorek, Robert Ryszard Chodorek, Alexander Yastrebov

**Affiliations:** 1 Department of Applied Computer Science, Faculty of Electrical Engineering, Automatic Control and Computer Science, Kielce University of Technology, Al. 1000-Lecia P.P. 7, 25-314 Kielce, Poland; a.chodorek@tu.kielce.pl (A.C.); a.jastriebow@tu.kielce.pl (A.Y.); 2 Institute of Telecommunications, Faculty of Computer Science, Electronics and Telecommunications, The AGH University of Science and Technology, Al. Mickiewicza 30, 30-059 Krakow, Poland

**Keywords:** field study, Internet of Things, unmanned aerial vehicle, weather monitoring, WebRTC

## Abstract

Thanks to IoT, Internet access, and low-cost sensors, it has become possible to increase the number of weather measuring points; hence, the density of the deployment of sources that provide weather data for the needs of large recipients, for example, weather web services or smart city management systems, has also increased. This paper presents a flying weather station that carries out measurements of two weather factors that are typically included in weather stations (ambient temperature and relative humidity), an often included weather factor (atmospheric pressure), and a rarely included one (ultraviolet index). In our solution, the measurements are supplemented with a visual observation of present weather phenomena. The flying weather station is built on a UAV and WebRTC-based universal platform proposed in our previous paper. The complete, fully operational flying weather station was evaluated in field studies. Experiments were conducted during a 6-month period on days having noticeably different weather conditions. Results show that weather data coming from the flying weather station were equal (with a good approximation) to weather data obtained from the reference weather station. When compared to the weather stations described in the literature (both stationary weather stations and mobile ones), the proposed solution achieved better accuracy than the other weather stations based on low-cost sensors.

## 1. Introduction

In the smart city concept, weather data from devices equipped with appropriate sensors—such as smartphones, cars, etc.—can be collected to complement information coming from classic, stationary weather stations. The high density of such ad-hoc-created weather stations and their mobility support provide high-density spatio-temporal weather data. This is the greatest advantage of this approach, especially in light of the short-distance validity (from less than one meter to hundreds of meters) of weather information gathered in the urban canopy layer [1]. The disadvantages are as follows: relatively low trustworthiness of the crowdsourced data [2], unknown accuracy of measurements, and uneven density of weather data sources (very dense in crowded places and during rush hours and sparse in rarely visited places).

For some time, weather stations were often built as Internet of Things (IoT) devices [3,4,5], which increases their availability. At the same time, the development of low-cost sensors, initially for the needs of the smartphone market, also entailed frequent use of such sensors in weather stations (e.g., [3,4,5,6,7,8,9,10]). This results in a reduction in the cost of weather stations, which indirectly contributes to an increase in the density of weather measurement points [7]. As was the case with ad-hoc smartphone-based weather stations, now the existence of multiple weather stations in a given area enable the collection of high-density spatio-temporal weather data from this area. Moreover, these low-cost (or relatively low-cost) weather stations usually measure and observe various parameters of weather [5,11] demanded by stations’ owners (or main users or administrators). As a result, a wide spectrum of weather measurements were carried out in a given area.

The authors believe that the use of Unmanned Aerial Vehicles (UAVs) based high-mobility weather stations and built with the use of low-cost sensors can overcome many of these disadvantages. Such flying weather stations are trusted sources of weather information with known accuracy and are available anytime (within the limitations resulting from the UAVs’ airworthinesses) and anywhere (within operating limitations resulting from the UAVs’ range and aviation law regulations). The term “anywhere” denotes not only horizontal distance from the starting point but also a vertical one. This could be the “cure” for the typical “illness” of low-cost weather stations often with compact building: all parameters are measured at the same height [12].

### Main Contributions and Organization of This Paper

While our previous paper was devoted to the UAV-based and WebRTC-based open universal framework for monitoring urban areas, this one is focused on a single purpose for this framework, namely a mobile weather monitoring system. The main contributions of this paper are as follows:

- The building of a mobile weather monitoring system based on the UAV and WebRTC-based universal platform, which offers the functionalities of an extended automatic weather station [12] (here, basic meteorological measurements—air temperature, relative humidity, and atmospheric pressure—are associated with the measurements of the ultraviolet index) as well as observations of present weather;
- Field experiments aimed at the evaluation of the flying weather station in different weather conditions;
- Comparative analysis of the results of measurements carried out by the proposed flying weather station and the results obtained by both reference devices and solutions known from the literature.

The rest of this paper is as follows. The Section 2 analyzes related work. The Section 3 presents materials and methods used during the building of the flying weather station. The Section 4 is devoted to experiments aimed at the evaluation of the proposed system. The Section 5 presents results of functional tests of the complete flying weather station at different times of the year and under different weather conditions and compares the results of measurements carried out by the proposed flying weather station to reference devices. The Section 6 discusses the results obtained versus those found in the available literature. The Section 7 summarizes our experiences.

## 2. Related Work

Weather stations either are stationary [3,4,5,6,7,8,11], mobile [8,9,10,13,14,15,16,17,18,19,20], or portable [21]. The mobile ones, especially UAV-based ones intended for weather sensing in the urban environment, usually do not alter but extend the use of stationary weather stations. This results in an increase in the density of the deployment of sources of weather data [22]. Amongst mobile weather stations, air ones are especially useful because data coming from air weather stations are usually a good supplement to ground station measurements [16]. Moreover, mobile weather sensing, including the ones based on UAVs, facilitate ad hoc coverage of places of interest. As an example, they enable one to measure weather factors and observe dangerous phenomena [22], such as storms or tornadoes [19,23], at close range.

During mobile weather measurements, different types of carriers are used beginning with satellites [24], through aerostats (balloons [20]), and ending with aerodynes. Satellites are too expensive to be popular carriers, balloons are uncontrollable, and manned aerodynes (airplanes or helicopters) are expensive (in terms of both equipment and flights) [24], require complex infrastructure, and will not undertake dangerous missions that could result in the destruction of the aircraft; UAVs are devoid of these disadvantages. They are very elastic in terms of tasks performed and generate the lowest costs. The first documented UAV-based weather sensing device was presented in this paper [20].

The UAVs are build as both fixed-wing ones [16,17,20,25,26,27] and rotorcraft ones (usually multicopters [8,9,10,13,14,17,18,28,29]). The biggest advantages of fixed-wing UAVs are typically longer flight time, greater range, and greater Region of Interest (ROI) [24]. However, these advantages are associated with a number of disadvantages, such as a large space needed for take-off and landing, difficulty in ensuring automatic take-off and large difficulties in ensuring automatic landing, less stability, higher minimum speed (which hinders a certain class of measurements), and limited maneuverability. This resulted in modern applications of air weather stations having fixed-wing UAVs being replaced by multirotor ones. The main advantages of multirotor UAVs are as follows: easy take-off and landing, the possibility of automatic take-off and landing, high maneuverability, compact size, and the ability to move in discontinuous routes and to achieve enhanced spatial resolution [24].

The use of UAVs has limitations in terms of energy consumption, endurance, and computation resources. The most important limitation is energy consumption, which can result in a relatively limited range of flight, which is a typical disadvantage of multirotor UAVs. This issue can be solved, for example, by UAV docking stations [30] or by custom planned routes [31]. The relatively small endurance of multirotor UAVs is caused mainly by one of their advantages, namely its compact size, and limits the applicability of UAV-based flying weather stations as well as strong winds that may result in the destruction of the UAVs. Limitations in terms of computation resources are negligible because single-board computers (SBCs), which deal with processing weather data, are generally the same as those used in stationary weather stations.

The dominant architectures of SBCs are two energy-saving ones: the Alf and Vegard’s RISC processor (AVR) [10,32,33,34,35] and the Advanced RISC Machine (ARM) [5,10]. However, other SBCs are also used. One of the rarer solutions of an SBC used in weather stations was the use of a doubled TrIMU navigation computer [16]. One of these computers was used for UAV navigation, and the other is dedicated to sensors and data transmission. In our implementation of a flying weather station, the Raspberry Pi 4B with Broadcom BCM2711 ARM processor was used, which is believed to have a good trade-off between energy consumption and computational power.

Weather stations typically provide measurements of atmospheric parameters, such as air temperature, relative humidity, ultraviolet (UV) radiation, wind velocity, atmospheric pressure, and rainfall [6]. While ambient temperature [3,4,5,6,7,8,9,10,13,14,16,20] and relative humidity [3,4,5,6,7,8,9,10,16,20] are typical measured parameters, atmospheric pressure was measured in about two-thirds of the analyzed stations [3,5,7,8,9,13,14,16,20], and one-third of them were capable of measuring some parameters of solar radiation (e.g., luminosity [8,10] and global solar radiation [6]). The UV index was measured by only one solution [9]. Our weather station was intended for measurements of both classic weather factors (temperature, humidity, and atmospheric pressure) and UV index (Table 1).

In an urban environment, cameras can be used, for example, for the surveillance of vehicles in intelligent transportation systems [36] and for the observation of weather events and phenomena [1]. General-purpose weather stations based on low-cost sensors typically do not use cameras for observation of present weather. A camera was not installed in all considered stationary weather stations [3,4,5,6,7,8] and in some UAVs [8,13,14,16]. Although in other UAVs, a camera was mounted [8,9,10], the video observations of present weather were not carried out, and the camera was considered to be used only for pilotage (or it was not used at all). However, the need of cameras for tasks other than pilot tasks is indicated in the literature [15]. Paper [37] describes an experiment in which a web camera was used for reading measurements from a mercury thermometer, and the possibility of using a camera for the visual observation of clouds was considered for future investigation. In our solution, visual data are integrated with data coming from sensors.

## 3. Materials and Methods

This section describes the UAV and WebRTC based universal framework, discusses the possibility of using this framework as a platform for building a flying weather station, discusses the weather factors selected to be measured by the flying weather station, presents sensors that will be used to measure these factors, and discusses the aspects of the observations of present weather.

### 3.1. The UAV and WebRTC-Based Universal Framework

The UAV-based and WebRTC-based universal framework [38] consists of an air station and a ground station. The biggest element of the air station is the IoT carrier, which is a quadcopter built on the Tarot FY650 isosceles frame. The IoT carrier was customized to accomplish tasks specific to monitoring urban and industrial areas, which resulted in good airworthiness and a maximum load of up to 1 kg believed to be enough to carry the IoT equipment. On board the IoT carrier, a Raspberry Pi 4 Model B single-board computer was mounted. To the SBC, both task-specific equipment (typically sensors, which should be selected in terms of a specific monitoring task) and framework-specific accessories (the Manta MM9359FS camera and the Waveshare SIM7000E positioning module) were connected.

**Figure 1:**
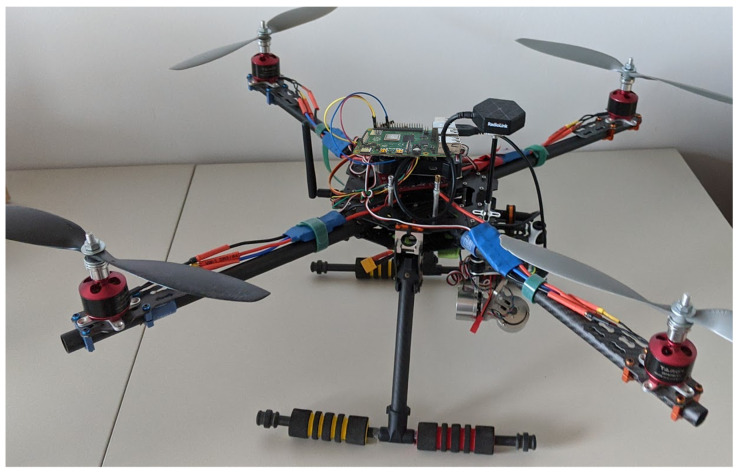
The air station of the UAV and WebRTC-based universal framework: the station stands on the laboratory table.

Figure 1 depicts the air station of the UAV and WebRTC-based universal framework ready for flight. On top of the station, the SBC is shown (green board). Under the right arm of the frame, the gimbal, on which the Manta MM9359FS camera is mounted, is observed (silvery cylinder).

The ground station is functionally and structurally divided into two devices [38]: the Command and Control Console (CCC) and the WebRTC Multimedia and Monitoring Station (WMMS). The first one is used for piloting the UAV, and the second one is used for the monitoring tasks. A FlySky FS-i6X console and a laptop computer were used, respectively, as the CCC and the WMMS.

The SBC gathers monitoring data and sends them to the WMMS part of the ground station. In order to improve the reliability of the remote pilotage, the bulk data traffic generated by the monitoring system and transmitted between the SBC and the ground station is separated from the control and telemetry traffic transmitted between the IoT carrier and the ground station. Due to the large amount of data produced by the 4K camera, the IEEE 802.11ac wireless local area network (WLAN) is used as the production network (the one intended for the transmission of monitoring data).

The monitoring software of the UAV and WebRTC based universal framework was built according to the Web of Things paradigm understood as the integration of smart things with the Web [39]. This software was originally developed for the purposes of e-health IoT [40], then adapted to air-to-ground transmissions from a drone [41], and finally modified for use in the framework [38]. The essence of this modification was described in [38].

### 3.2. Analysis of the Possibility of Using the Framework Hardware as a Platform for Building Flying Weather Stations

When comparing related work with the UAV-based and WebRTC-based universal frameworks, great similarities between the framework and the described solutions can be observed in terms of the hardware used. The framework, as with most of the presented solutions for flying weather stations [8,9,10,13,14,17,18], uses a multicopter as an IoT carrier. This medium-size quadcopter that weighs less than 2 kg is between the two multicopter carriers of weather stations known from the literature: the light-weight Parrot AR.Drone 2.0 used in [10] and the heavy DJI Matrice 600 used in [9]. It is worth remarking that the IoT carrier included in the framework has the same maximum load as the Carolo T200, which is twice as heavy, used in [16] for carrying meteorological equipment.

The SBC, used in the framework as the IoT hub, is the multi-purpose Raspberry Pi, which is also used in weather stations (e.g., [5,10]). Monitoring data are transmitted to a remote logger, which is the WMMS by default. Since communication is carried out with the use of the Message Queue Telemetry Transport (MQ Telemetry Transport, MQTT) protocol, remote loggers can also be located at MQTT subscribers of particular data. In such a situation, the MQTT works as a relay broker, which intermediates in data transmissions. To communicate between the air station and the ground station, IEEE 802.11 WLAN is used, which is the same as in some weather stations known from the literature (e.g., [3,4,5,10]).

As a result, there is no problem for the hardware to use the UAV and WebRTC based universal framework as a platform for building flying weather stations. The use of this framework for prototyping a weather station requires the selection of sensors in terms of their usability for a given weather station and then their mechanical and electrical integration within the framework.

### 3.3. Selection of Weather Factors Measured by the Flying Weather Station

The World Meteorological Organization (WMO) in [1], devoted to meteorological observations at urban sites, include guidances on measurements of several weather factors. The factors are as follows: temperature, atmospheric pressure, humidity, wind speed and direction, precipitation, radiation, sunshine duration, visibility and meteorological optical range, evaporation, soil moisture, and atmospheric composition. Some of the above weather factors, such as precipitation or soil moisture, cannot be easy measured by a UAV-based weather station during a flight. However, they can be measured and recorded by autonomous IoT devices and transmitted to the arriving flying weather station. Although UAV and WebRTC based platforms enable cooperation with remote, autonomous IoT devices, the air station act as a communication hub rather than the weather station during such cooperation; thus, we decided to omit this functionality in this pilot study. Other omitted weather factors are the ones that are not measured by popular stationary weather stations, mainly because of difficulty in receiving accurate reference measurements to carry out proper evaluation. Due to the specificity of the urban area, weather factors can vary in very short distances; therefore, experimental evaluation should be conducted in close proximity to the reference weather station.

As a result, we decided to measure four weather factors. Three of them were the most popular classic ones:

- Ambient temperature, measured by all solutions listed in Table 1 [3,4,5,6,7,8,9,10,13,14,16,20,21,33];
- Relative humidity, measured by all solutions listed in Table 1 that used low-cost sensors [3,4,5,6,7,8,9,10,16,20,21,33];
- Atmospheric pressure, measured by two-thirds of the solutions listed in Table 1 [3,5,7,8,9,13,14,16,20,33].

The fourth one was the UV index, introduced by the World Health Organization (WHO) as the global solar measure of exposure to solar ultraviolet radiation [42]. This weather factor was measured by only one weather station amongst those listed in Table 1. Nonetheless, knowledge about the current value of the UV index is important from the public health point of view. As indicated in paper [43], because of relatively high-speed variations of the UV index in time and space, current measurements of this factor should be made available to the public.

### 3.4. Sensors Applied in the Flying Weather Station

As an atmospheric pressure sensor, the three-in-one (air pressure, temperature, and humidity) Bosch Sensortec BME280 was selected. It is a popular and low-cost sensor that is applicable to smartphones and Arduino boards, as well as to smartwatches and smartbands.

According to the BME280 sensor manual, the nominal weight of this sensor is 1.7 g. Atmospheric pressure measurements are performed according to the sensor’s specification with an accuracy of ±0.12 hPa, temperature measurements with an accuracy of ±0.5 ${}^{\circ }$C, and humidity measurements with an accuracy of ±3%. The measured energy consumption (when pressure, temperature, and humidity measurements were simultaneously carried out) during its work in the fully operational flying weather station was 340 μA.

The current practice during sensor selection is to select one common sensor for multiple weather factors measurements. As an example, triple measurements were used when the BME280 sensor was used [5,7,8,9] or double measurements were used when a temperature and humidity sensor was used [3,4,6,8,10]. However, BME280 was not able to achieve satisfactory accuracy of temperature measurements. Thus, measurement HTU21D was selected for carrying out temperature and humidity measurements in the flying weather station due to having the best quality humidity information. The nominal accuracy of HTU21D during temperature measurements is ±0.3 ${}^{\circ }$C, and during humidity measurements it is ±2%. The nominal weight of the HTU21D, given by the manual, is 1.8 g. The current consumed by the HTU21D during operation in the flying weather station was 420 µA.

As UV index sensors, Silicon Laboratories SI1145 and the Vishay Semiconductors VEML6070 were selected. Silicon Laboratories SI1145 multi-channel digital sensor board is a low power, reflectance-based light sensor. This general-purpose sensor is devoted to measuring infrared proximity, UV index, and ambient light. It is used as an ambient light sensor in handsets and notebooks, as an infrared sensor in smoke detectors, and—last but not least—as a UVI sensor across a wide range of devices, including weather stations. The nominal accuracy of the SI1145 during UV index measurements is not included in the sensor’s documentation (the same is true for the VEML6070 UV index sensor). Its nominal weight is 1.4 g. Its current consumption measured during the study in the fully operational flying weather station was 250 µA.

Vishay Semiconductors VEML6070 is used in outdoor sport handheld products. This sensor measures UV radiation only, and the values of the UV index must be calculated from UV radiation by the monitoring application. VEML6070 is the most light-weight sensor among the selected ones and one that saves the most energy. It weighs only half a gram, nominally, and the current consumed by VEML6070 during operation in the flying weather station was 110 µA.

### 3.5. Observations of Present Weather

**Figure 2:**
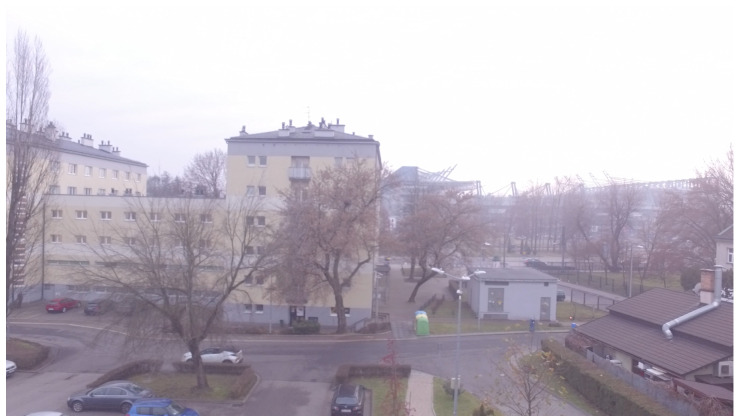
The view from the flying weather station.

The authors of [1] mentions observations of present weather (including weather events and phenomena: rime, snow, ice, fog, lightning, etc.) and observations of clouds as important supplements to the measurements of weather factors. In an urban environment, due to the plethora of intensive light sources and the polluted atmosphere, visual observations of clouds are difficult, but there is nothing to prevent (except the lack of proper equipment) the observation of present weather. However, although some of the UAV-based weather stations included in Table 1 have installed UAV cameras, none of the items in this table gives a visual observation as a feature of a reported solution. In the case of the proposed flying weather station, for visual observation of present weather, the 4K gimbal UAV camera was used. An example of a visual observation made with the operating flying weather station is shown in Figure 2.

In contrast to the framework [38], moving pictures from the camera contain no metadata describing the context of the data, but full-fledged data are on par with the data coming from the sensors. Video frames are integrated with other data and metadata and are linked to other data, which can be valuable for further analysis. Due to the use of the MQTT, a subscriber of meteorological data can be situated in a remote location, far away from the place of data gathering and visualize online the current meteorological situation on a dashboard or use stored data for retrospective analysis. In the case of the online assessment of present weather, clouds, or currently appearing meteorological events and phenomena, the use of the framework’s option of the full-size video displayed on an external monitor may be very helpful for analysts.

## 4. Experiments

Experiments on a flying weather station differ from ground-based experiments mainly by their horizontal and vertical mobility. Due to the fact that the described experiments were focused on the evaluation of a proposed solution, vertical mobility of the weather station was not tested.

This section describes the location of the experiments, the assignment of sensor attributes, system integration, flying days, and flight sessions.

### 4.1. The Location of Experiments

The flying weather station was evaluated in experiments carried out in a rectangular parking lot (70 m × 70 m) located inside the campus of AGH University of Science and Technology (Krakow, Poland). Flights over this parking lot are limited by university buildings, buildings owned by external entities, trees on the east side, and standing lighting columns in the car park area. These obstacles were taken into account during the planning of the automatic flight of the IoT carrier. However, the altitude of the flight was high enough to allow the weather station to fly over obstacles located directly over the parking lot, which was the location of the experiments.

The reference weather station was located on the roof of one of the university buildings. The straight horizontal distance between the flying weather station and the reference one was from about 100 m to about 200 m. This was small enough to assure the same values of weather factors.

### 4.2. Assignment of Attributes

For the experiments, each sensor selected for use in the flying weather station had one or more attributes assigned, thereby assigning roles played in the flying weather station. To the Bosch Sensortec BME280, two attributes were assigned: the primary atmospheric pressure sensor and the secondary humidity sensor. To Measurement HTU21D, two attributes were also assigned: the primary temperature sensor and the primary humidity one. The Silicon Laboratories SI1145 and the Vishay Semiconductors VEML6070 received the attributes of the primary and secondary UV index sensors, respectively.

Only information coming from the primary and secondary sensors can be considered as public. Thus, although BME280 was also used as a temperature meter, temperature data coming from this sensor were considered as private and unrelated to the performed task. The same applies to the UAV’s telemetry data. As an example, atmospheric pressure and temperature measurements coming to the WMMS from the MEAS Switzerland MS561 barometric pressure sensor, which is the main part of the barometric altimeter of the IoT carrier, were also filtered out as private ones.

### 4.3. System Integration

The use of sensors in the flying weather station requires both hardware and software integration. Hardware integration involves the mechanical and electrical connection of a sensor to the SBC. All selected sensors are digital ones, and they were electrically connected through the Inter-Integrated Circuit (I${}^{2}$C) bus to the General Purpose Input-Output (GPIO) interface of the SBC.

Software integration involves writing the initialization and sensor service modules and their integration with the core software. While the core software takes care of communication, positioning, and video from the carrier’s camera, the newly written modules deal with the sensors connected to the SBC. The initialization module sets the SBC’s GPIO pins to a proper mode, as well as sensors; it then sets up sensors relative to the required resolution and sampling rate. The sensor service module cyclically pools sensors in order to collect weather measurement data.

### 4.4. Selection of Flying Days

To assure a variety of weather conditions suitable for the experimental evaluation of the flying weather station, we selected five days from a 180-days base interval, from early summer to late autumn. We chose two days from May to June, one from September, and two from November. Days in pairs at the beginning and the end of the time interval were separated by an interval of one week.

The May–June period in Poland is when spring turns into summer, with intensive solar irradiation, high temperatures, and lower humidity (although it depends on the day because this is also the end of the period of May rains). September is when summer turns into autumn, characterized by medium insolation level, medium humidity, and medium temperature. November is late autumn, with a lower level of sunlight (cloudy days and the sun is low in the sky), high humidity, and relatively low temperatures. At the end of November, temperatures below zero and snow are not uncommon.

### 4.5. Flight Sessions

The field experiment was organized in flight sessions. Each flight session consisted of five flights in a row that are separated by maintenance break if needed, intended for battery replacement. Depending on weather conditions, the battery was replaced after every flight or every two flights.

There were two terms of flight sessions. The morning flight session beginning at about 6:30 a.m. and lasts to about 7:50 a.m. The midday flight session, as the name suggests, was organized sometime around midday, i.e., from 11 a.m. to 2 p.m.

The flight session on day 1 was conducted at the end of May, in the midday. Exactly a week later, at the beginning of June, a morning flight session was performed (day 2). The flight session on day three was the morning one, conducted in the middle of September. Two midday flight sessions were carried out in the second half of November, with an interval of a week (day four and day five, respectively).

During a single flight, the weather station that flew at the operating altitude (15 m above ground level) crossed the test parking lot, then flew a few meters along the parking boundary, and crossed the parking lot again. This was repeated until the weather station flew to the end of the parking lot. Then, it crossed the parking lot diagonally in order to return to the starting point. In the sections that crossed the parking lot, every 5 m measuring points were established. The weather station hovered over successive measuring points for 2 s. The last most stable measurement of each hovering phase was filtered as public data and taken for further processing.

## 5. Evaluation

In this section, the results of field experiments aimed at the evaluation of the flying weather station are presented. Experiments were carried out in different weather conditions and in five separate days (from day 1 to day 5). The results of measurements of four weather factors (atmospheric pressure, temperature and humidity, and UV index) carried out by the weather station were compared with the corresponding results obtained with the use of reference devices (the SBS-WS-400 weather station and barometric altimeter).

Since air pressure and temperature change with altitude, all measurements were carried out at the altitude of the reference weather station. No significant differences between the results of measurements carried out on the fly and when hovering were observed.

### 5.1. Atmospheric Pressure Sensing

The flying weather station was tested in field experiments carried out in different weather conditions. Table 2 shows minimum, maximum, and mean value of absolute atmospheric pressure measured by this station during five flight sessions that took place on five separate days from the end of May to the end of November. The mean relative pressure, reduced to sea level, was calculated and presented in Table 2 as well. As the basis for calculations, the temperatures measured by the primary temperature sensor of the flying weather station (Section 5.2) and the height above sea level of 220 m were used. In the experiments, the weather station flew at an altitude of 15 m above the test site elevation (about 205 m above sea level).

The results of measurements carried out by the primary sensor of the flying weather station (Table 2) show that during three of the five flight sessions (day 1, 4, and 5), low atmospheric pressure (mean relative atmospheric pressure less than or equal to the normal one, i.e., 1013.25 hPa) was registered. The lowest air pressure (981.8 hPa) was observed during the midday flight session carried out in the second half of November (on day 4). Maximum pressure, which was then registered (985.0 hPa), was lower than the minima registered on other days. Moreover, on day 4, the lowest mean pressure occurred (absolute: 983.5 hPa, relative: 1010 hPa), and the greatest difference between the minimum and maximum atmospheric pressure (3.2 hPa), observed during a single flight session, was then recorded.

The smallest difference between the minimum and maximum absolute atmospheric pressure (0.6 hPa) occurred during the morning flight session conducted at the beginning of June (on day 2). This small difference was associated with a large stability of measured values, which found expression in a margin of error bound to the order of a few pascals. This is visible in Table 2 as the margin of error is equal to zero (0.04 hPa rounded to the nearest tenth). Day 2 was also one of the two days (the other was day 3) with a high atmospheric pressure, i.e., with mean relative atmospheric pressure greater than 1013.25 hPa. On this day, the highest mean air pressure was observed (absolute: 992 hPa, relative: 1017.2 hPa), and the highest maximum pressure was observed as well. The minimum one (991.7 hPa) was greater than maximum values registered on the other four days.

Atmospheric pressure measurements made by the flying weather station were compared with two independent reference measurements performed by the MEAS Switzerland MS561 barometric pressure sensor (working as the barometric altimeter on the IoT carrier) and by the Steinberg Systems SBS-WS-400 weather station on the roof of the nearby building, 15 m above ground level (Table 2). Although these three devices have different nominal measurement accuracies (BMP280: ±1 hPa; MS5611: ±1.5 hPa; SBS-WS-400: ±5 hPa), no differences (with an accuracy of ±0.2 hPa) were observed between the corresponding results of the measurements. What is especially important is the compliance of the flying weather station measurements with the measurements performed by the MS5611, which is considered to be well factory calibrated.

**Figure 3:**
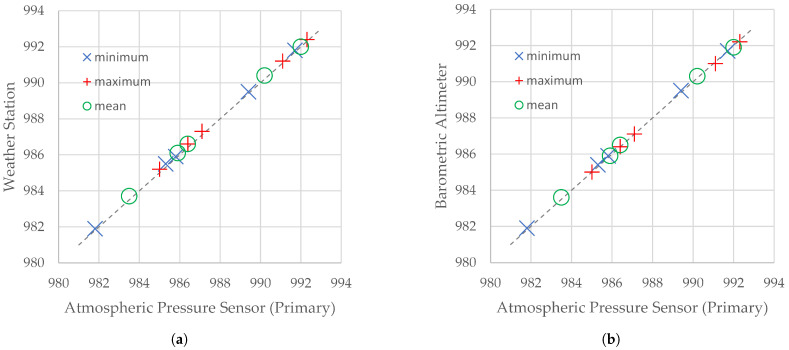
Scatter plots for statistics of atmospheric pressure measured by a reference device and the primary atmospheric pressure flying weather station. Reference device: (**a**) the SBS-WS-400 weather station; (**b**) barometric altimeter.

Figure 3 shows scatter plots drawn for the statistics of atmospheric pressure included in Table 2. Markers denote maximum, minimum, and mean values of the atmospheric pressure obtained during the same flight sessions by a reference device and the flying weather station. On both graphs, markers are spread across the diagonal of the plot (dotted line), which proves that differences between the statistics of atmospheric pressure included in Table 2 are very small.

### 5.2. Temperature and Humidity Sensing

The flying weather station was tested in field experiments in terms of temperature and humidity measurements. The results of experiments carried out on day 1 to day 5 are summarized in Table 3 and Table 4.

During the midday flight session conducted at the end of May (day 1), the difference between the minimum measured air temperature and the maximum one was about 6 ${}^{\circ }$C, and humidity was under 50%. These were the greatest differences in temperature observed during the flight sessions presented in Table 3 and the lowest value of humidity measurements shown in Table 4. A week later, at the beginning of June (day 2), the proximity of the longest day of the year meant that the temperatures were greater than measured on day 1, and the higher humidity indicated that data were gathered during the morning session. Autumn measurements (days 3, 4, and 5) were characterized by lower and lower temperatures and increasing humidity. Mean temperature measured by the primary sensor of the flying weather station during the morning flight session in the middle of September was 16.2 ${}^{\circ }$C, and mean humidity was 75%. After 6 weeks, the temperature had fallen to 2.9 ${}^{\circ }$C, measured during midday by the same sensor, and mean humidity increased to 95%.

The results of the measurements of temperature and humidity carried out with the use of the primary sensor of the flying weather station were compared with corresponding results obtained from the reference weather station (Steinberg Systems SBS-WS-400) and the reference temperature sensor (the MEAS Switzerland MS561 sensor working as the barometric altimeter on the IoT carrier). The weather station’s manual specifies that the accuracy of the temperature measurement is ±1 ${}^{\circ }$C. The nominal accuracy of the MEAS Switzerland MS561 is ±0.8 ${}^{\circ }$C. As is observed in Table 3 and Table 4, the results coming from these three devices are, in general, comparable.

**Figure 4:**
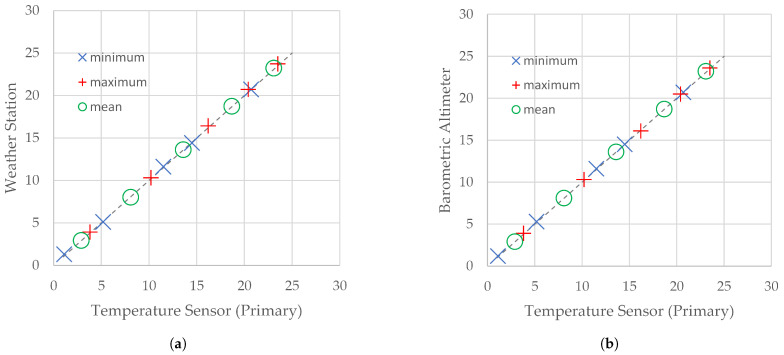
Scatter plots for statistics of temperature measured by a reference device and the primary temperature sensor of the flying weather station. Reference device: (**a**) the SBS-WS-400 weather station; (**b**) barometric altimeter.

Despite the fact that sensors on board the flying weather station are exposed to sunlight and sensors of the reference weather station are hidden in the instrument shelter, extrema and means of temperatures measured by the primary sensor (HTU21D) did not differ significantly from the corresponding statistics calculated on the basis of reference measurements (Figure 4). Extrema and means are approximately the same as the reference measurements carried out by both the SBS-WS-400 weather station (typically with an accuracy of up to 0.2 ${}^{\circ }$C) and the MS561 reference temperature sensor (with an accuracy of up to 0.1 ${}^{\circ }$C). This results in the conclusion that neither wind cooling nor airflow cooling caused by station movement was sufficient to cause systematic error.

It should be noted that the difference between maximum temperatures registered by the primary sensor of the flying weather station and by the reference weather station exceeded 0.2 ${}^{\circ }$C only once on day 1 (0.3 ${}^{\circ }$C). Since the maximum temperatures registered by sensors on board the flying weather station are almost the same (20.4 ${}^{\circ }$C and 20.5 ${}^{\circ }$C) and smaller than the maximum temperature registered by the reference weather station (20.7 ${}^{\circ }$C), this larger-than-usual difference was caused, in our opinion, by local circumstances rather than by a feature of the WebRTC-based IoT.

**Figure 5:**
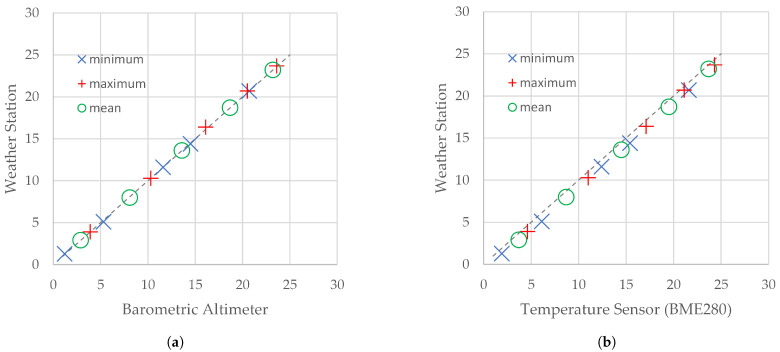
Scatter plots for statistics of temperature measured by the SBS-WS-400 reference weather station and the (**a**) barometric altimeter and (**b**) the BME280.

For the sake of comparison, we drew scatter plots for the SBS-WS-400 and barometric altimeter (Figure 5a) and for the SBS-WS-400 and the BME280 (Figure 5b). As shown in Figure 5a, the are only slight differences between the sources of reference measurements. Figure 5b, in turn, clearly shows the systematic error.

**Figure 6:**
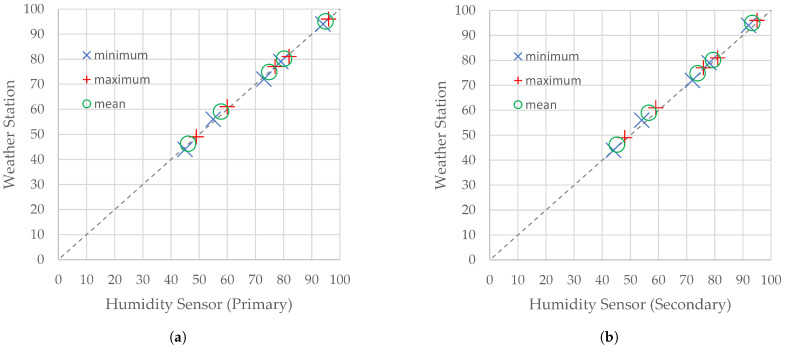
Scatter plots for statistics of humidity measured by the SBS-WS-400 reference weather station and (**a**) the primary humidity sensor of the flying weather station and (**b**) the secondary one.

In the case of measurements of humidity, shown in Table 4, extrema of measurements obtained from the primary sensor of the flying weather station and from the reference weather station have an accuracy of up to ±1 percentage point. The corresponding extrema of measurements obtained from the primary sensor and the secondary one result in an accuracy of up to ±2 percentage points. Differences between mean relative humidities obtained by the primary humidity sensor and by any other source shown in Table 4 approached up to 1.2 percentage points. A comparison of the statistics of humidity measured by the reference weather station and the statistics obtained by the primary humidity sensor and the secondary one is depicted in Figure 6.

### 5.3. UV Index Sensing

The Silicon Laboratories SI1145 sensor working as the primary UV index sensor and the Vishay Semiconductors VEML6070 sensor working as the secondary one were tested in field experiments. Statistical results of these experiments are presented in Table 5.

During the midday flight session on day 1 (the end of May), the UV index measured by the primary sensor of the flying weather station reaches 7.21 (7.02 on average). Measurements carried out at the beginning of June during the morning flight session (day 2) show a significant increase in UV index compared to the results of experiments conducted a week earlier. Despite earlier hours, the maximum measured UV index and the mean one was more than 10% greater than the respective statistics of the UV index obtained for day 1, and the minimum one was nearly 10% greater than the minimum of UV indexes collected on day 1. This was caused by it being closer to the longest day of the year. Autumn measurements (days 3, 4, and 5) show a sharply falling insolation, expressed in the fall of the UV index. As a result, the UV index measured by the primary sensor dropped from 7.93 on average (morning in early June), through 4.51 on average (September morning), to 0.76 on average (midday in late November). The mean UV index, determined as the integral part of the mean measured UV index, shows this tendency more strongly: from seven at the turn of spring and summer to four in early fall and to zero at the turn of autumn and winter.

A comparison of the primary UV index sensor with the secondary one shows no differences in the UV index (determined from the mean), although differences between corresponding statistics of measured UV indexes are observed. In the collected dataset, the extrema of measurements performed with the use of the Vishay Semiconductors VEML6070 were always (maxima) or almost always (minima) greater than the corresponding minima and maxima of measurements carried out by using the Silicon Laboratories SI1145. In the case of average measurements, this regularity is absent. The longer integration time of the secondary sensor results in a narrower margin of error.

**Figure 7:**
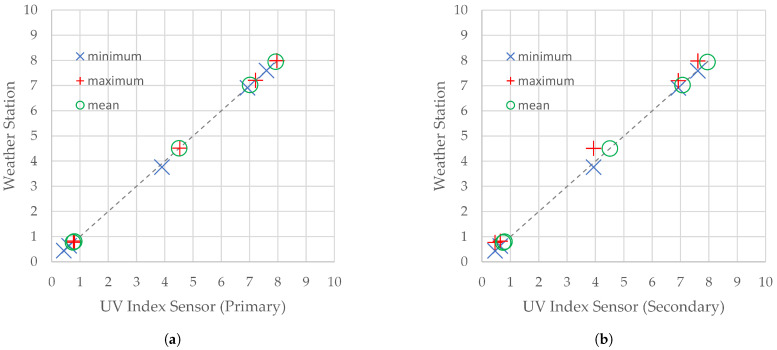
Scatter plots for statistics of UV index measured by the SBS-WS-400 reference weather station and (**a**) the primary UV index sensor of the flying weather station and (**b**) the secondary one.

The values of the mean UV index obtained from both sensors of the flying weather station and from the reference weather station are exactly the same. Measured UV indexes, which are the basis for determining the mean UV index, are also comparable (Figure 7). Although the secondary sensor (VEML6070) tends to slightly overstate the maximum value, it does not overstate the arithmetic mean (Figure 7). This indicates that the measurement is not burdened with a systematic error.

The differences between results collected in Table 5 do not exceed 0.02 and 0.05 when comparing, respectively, the SI1145 and the VEML6070 sensors with SBS-WS-400. The only exceptions are the minimum values measured by the flying weather station on day 3, which significantly differed from the corresponding value measured by the reference weather station (3.90/3.93 vs. 3.76). Considering that the SBS-WS-400 weather station was placed about 100 m from the boundary of the test site, the differences in maximum value are not as large, and the observed differences in mean were within the limits of statistical error; this difference is caused by local phenomena rather than by features of the flying weather station.

## 6. Comparison with Related Work

As was presented in the previous section, this flying weather station was the subject of field experiments. These were functional tests, aimed at checking this station in its work environment in different weather conditions. To assure a variety of weather conditions during field tests, experiments took place during three different seasons (at the turn of spring into summer, in early fall, and at the turn of autumn into winter). This allowed the flying weather station to be successfully tested in a wide range of the following factors:

- Atmospheric pressures (from 981.8 hPa to 992.3 hPa of absolute pressure);
- Air temperatures (from 1.1 ${}^{\circ }$C to 23.5 ${}^{\circ }$C);
- Relative humidities (from 45% to 96%);
- UV indexes (from 0 to 7 and measured UV indexes, including tenths, from 0.43 to 7.21).

Solutions from related work reports five tests carried out in different atmospheric pressures. The range of these differences were (in ascending order) 1 hPa [9], 4 hPa [7], 7 hPa [16], 10 hPa [5], and 20 hPa [3], and the two largest pressure ranges occurred during the tests of stationary weather stations. This shows that the proposed weather station passed the functional tests in a relatively large range of pressures (10.5 hPa), which was wider than those occurring in the tests of other mobile weather stations (1 hPa [9] and 7 hPa [16]) and close to the median of ranges occurring in the tests of stationary ones.

The temperature of 1.1 ${}^{\circ }$C was the minimum that occurred during the functional tests of the flying weather stations. Compared to related work, only two papers out of ten show a lower minimum temperature of −20 ${}^{\circ }$C [5] measured during tests of a stationary weather station and −2.5 ${}^{\circ }$C [16] measured during tests of a mobile weather station flying at altitude of 800 m. This places our station within the solutions that were best tested in low temperatures. The maximum temperature that was close to 24 degrees Celsius places it around the median of upper limits of tested temperatures. The 23 ${}^{\circ }$C range of temperature that occurred during tests again places our station in the group of the best tested solutions when compared to related work. Although two reported solutions were tested in wider ranges of temperatures (37 ${}^{\circ }$C [8] and 47 ${}^{\circ }$C [5]), there are weather stations with 20 times narrower [9] or 10 times narrower [3,4,16] range of temperatures that occurred during their functional tests.

The lower limit of the range of humidities that occurred during functional tests (45%) was the median of related work. The lower limit of the range of humidities (96%) was the best equally placed with the solution described in [6] (97%) and [8] (95%). In general, the flying weather station passed functional tests in a very wide range of humidities (51%). Only one weather station was tested in wider range of humidities (85% [8]), one in almost the same width of range (52%), and the rest was tested in ranges that were from 17 times [3] to 4 times [7] narrower.

Ranges of atmospheric pressure, ambient temperature, and relative humidity that occurred during functional tests of solutions known from the literature are included in Table 6.

Table 7 presents the absolute error of values measured by primary sensors of the flying weather station, assuming that the reference weather station was the source of exact values. These errors were compared with errors obtained in functional tests of solutions known from related work (Table 8). The maximum absolute error obtained by the flying weather station during atmospheric pressure measurements was 0.2 hPa. This is about four times larger than the maximum absolute error of tests of weather stations equipped with the non-low-cost Sensortechnics sensor [16]. The solution [3], which used the BMP180 low-cost sensor, achieved a maximum relative error of 1.23% at the range of 1003 hPa to 1023 hPa, which provides 12.34 hPa to 12.58 hPa of maximum absolute error.

The maximum absolute temperature error of 0.3 ${}^{\circ }$C that occurred during day 1 was discussed in the previous section and considered as caused by local phenomena and not related to the operation or construction of flying weather station. Thus, the maximum absolute error is 0.2 ${}^{\circ }$C. Paper [16] reports a temperature error of ±0.6 K (0.6 ${}^{\circ }$C) when measurements with the use of a custom sensor were carried out. In paper [3], measurements with the use of the DHT11 low-cost sensor produced a maximum relative error of 1.4%, which at the range of 27.6 ${}^{\circ }$C to 30 ${}^{\circ }$C produced approximately 0.4 ${}^{\circ }$C of maximum absolute error. The average relative error calculated from measurements of the primary temperature sensor is 0.02% to 0.55% (Table 7). The average relative error of temperature measurements reported in [10] is 3.71% (Table 8). The weather station [10] used the RHT03 low-cost sensor for measurements of temperature and humidity.

In the case of humidity measurements carried out by the primary sensor, the maximum absolute error is 2% (Table 7). The same value (2%) was achieved by the weather station [16] that used the Vaisala Intercap sensor. The weather station [3] that used DHT11 for humidity measurements obtained a maximum relative error of 3.85%, which at a range of 75% to 78% produces about 3% of maximum absolute error. The average relative error calculated for the primary humidity sensor is 0.11% to 1.36% (Table 7). The weather station [10] obtained an average relative error of 1.65% (Table 8).

As is shown in Table 7, UV index measurements carried out by the primary sensor had the maximum absolute error of 0.14. The average relative error was 0.22%. Both errors relate to the measurements performed on day 3.

## 7. Conclusions

The flying weather station was built by using the UAV and WebRTC-based universal framework intended for the fast prototyping of different types of monitoring systems. This framework was reported in our previous work. The flying weather station performs measurements of weather factors, including atmospheric pressure, temperature, and humidity. What distinguishes this solution from similar works is mainly the following: measurement of UV index, the use of the carrier’s camera for observations of present weather, protection of measurements at the cost of video observations, and automatic software update.

The measurements of UV index were carried out by a minority of related work. Stationary weather stations typically are not equipped with cameras, and a UAV’s cameras are usually used for piloting the UAVs. Our solution treats the camera as equal to the sensors, and video information from the camera is integrated with data from the sensors. Measurements, observation, time, and location are integrated in a common context, and visual context is fully integrated with other data and metadata. This requires congestion control of video data and protection of measurements, which should be reliably transmitted even at the cost of video observations. This is achieved by the use of WebRTC technology. WebRTC also assures automatic software update with a suitable security level based on cryptographical protection of both data and video.

The flying weather station was tested in different weather conditions. Thanks to an experiment that stretched over time from May to June to the end of November, it was possible to carry out functional tests in wide ranges of air pressures, temperatures, and humidities. As a result, it was one of the best tested solutions in terms of the variety of atmospheric conditions when compared to the literature. Functional tests show that this flying weather station was able to achieve accuracy of weather measurements comparable with the stationary SBS-WS-400 weather station for wide range of meteorological parameters.

The simple and effective structure, the good cooperation of the monitoring software with sensors, and the proper selection of sensors caused the following result: when compared to weather stations known from the literature, the proposed flying weather station always achieved better accuracy than other weather stations based on low-cost sensors.

Future investigations will focus on the use of onboard artificial intelligence hardware accelerator provided by the UAV and the WebRTC-based framework to process both video information and data coming from the sensors.

## Figures and Tables

**Table 1:** Measured weather factors.

Paper	Station	Measured Weather Factors	Camera
[6]	stationary	temperature, humidity, global solar radiation	not mounted
[9]	mobile	temperature, humidity, pressure, UV index, ambient light	used only for pilotage
[13,14]	mobile	temperature, pressure	not mounted
[3]	stationary	temperature, humidity, pressure, rain	not mounted
[7]	stationary	temperature, humidity, pressure	not mounted
[4]	stationary	temperature, humidity, wind speed	not mounted
[5]	stationary	temperature, humidity, pressure, rain	not mounted
[16]	mobile	temperature, humidity, static and dynamic pressure	not mounted
[10]	mobile	temperature, humidity, solar radiation (luminosity only)	not used
[20]	mobile	temperature, humidity, pressure, air speed, vertical velocity	not mounted
[8]	stationary	temperature, humidity, rain, solar radiation (luminosity only)	not mounted
[8]	mobile	temperature, humidity, pressure	not mounted
[33]	mobile	temperature, humidity, pressure	not used
[21]	portable	temperature, humidity, solar radiation	not mounted
this paper	mobile	temperature, humidity, pressure, UV index	weather observations

**Table 2:** Atmospheric pressure (in hPa).

Day	Atmospheric Pressure Sensor (Primary)				Barometric Altimeter			Weather Station		
	Absolute Pressure			Relative Pressure	Absolute Pressure			Absolute Pressure		
	Min	Max	Mean	Mean	Min	Max	Mean	Min	Max	Mean
day 1	985.3	986.4	985.9 ± 0.1	1011.4	985.4	986.4	985.9 ± 0.1	985.5	986.6	986.1 ± 0.1
day 2	991.7	992.3	992.0 ± 0.0	1017.2	991.7	992.2	991.9 ± 0.0	991.8	992.4	992.0 ± 0.0
day 3	989.4	991.1	990.2 ± 0.1	1016.3	989.5	991.0	990.3 ± 0.1	989.5	991.2	990.4 ± 0.1
day 4	981.8	985.0	983.5 ± 0.1	1010.0	981.9	985.0	983.6 ± 0.1	981.9	985.2	983.7 ± 0.1
day 5	985.8	987.1	986.4 ± 0.1	1013.5	985.9	987.1	986.5 ± 0.1	985.9	987.3	986.6 ± 0.1

**Table 3:** Ambient temperature (in ${}^{\circ }$C).

Day	Temperature Sensor (Primary)			Barometric Altimeter			Weather Station		
	Min	Max	Mean	Min	Max	Mean	Min	Max	Mean
Day 1	14.5	20.4	18.7 ± 0.4	14.5	20.5	18.7 ± 0.1	14.4	20.7	18.7 ± 0.3
Day 2	20.7	23.5	23.1 ± 0.2	20.7	23.6	23.2 ± 0.1	20.7	23.7	23.2 ± 0.2
Day 3	11.5	16.2	13.6 ± 0.4	11.6	16.1	13.6 ± 0.2	11.6	16.4	13.6 ± 0.3
Day 4	5.2	10.2	8.1 ± 0.3	5.3	10.3	8.1 ± 0.1	5.1	10.3	8.0 ± 0.3
Day 5	1.1	3.8	2.9 ± 0.2	1.2	3.9	2.9 ± 0.1	1.3	3.9	2.9 ± 0.2

**Table 4:** Relative humidity (in %).

Day	Humidity Sensor (Primary)			Humidity Sensor (Secondary)			Weather Station		
	Min	Max	Mean	Min	Max	Mean	Min	Max	Mean
Day 1	45	49	46.1 ± 0.2	44	48	45.2 ± 0.2	44	49	46.2 ± 0.2
Day 2	55	60	57.8 ± 0.3	54	59	56.6 ± 0.3	56	61	59.0 ± 0.2
Day 3	73	77	74.9 ± 0.2	72	76	73.9 ± 0.2	72	77	74.8 ± 0.1
Day 4	79	82	80.2 ± 0.3	78	81	79.4 ± 0.2	79	81	80.1 ± 0.2
Day 5	94	96	94.9 ± 0.3	92	95	93.4 ± 0.2	94	96	95.0 ± 0.1

**Table 5:** Statistics of UV index measurements.

Day	UV Index Sensor (Primary)				UV Index Sensor (Secondary)				Weather Station			
	Measurements			Index	Measurements			Index	Measurements			Index
	Min	Max	Mean	Mean	Min	Max	Mean	Mean	Min	Max	Mean	Mean
Day 1	6.93	7.21	7.02 ± 0.03	7	6.92	7.24	7.07 ± 0.01	7	6.91	7.20	7.02 ± 0.02	7
Day 2	7.60	7.97	7.93 ± 0.03	7	7.61	8.01	7.95 ± 0.01	7	7.59	7.98	7.94 ± 0.02	7
Day 3	3.90	4.53	4.51 ± 0.02	4	3.93	4.54	4.51 ± 0.02	4	3.76	4.51	4.50 ± 0.01	4
Day 4	0.62	0.81	0.80 ± 0.02	0	0.64	0.82	0.79 ± 0.01	0	0.63	0.82	0.80 ± 0.01	0
Day 5	0.43	0.77	0.76 ± 0.02	0	0.46	0.78	0.75 ± 0.01	0	0.44	0.77	0.76 ± 0.01	0

**Table 6:** Experiments carried out in related work.

Paper	Station	Test Type	Ranges		
			Pressure	Temperature	Humidity
[6]	stationary	functional test	N/A	6 ${}^{\circ }$C–24 ${}^{\circ }$C	45–97%
[9]	mobile	functional test	1014 hPa–1015 hPa	19.4 ${}^{\circ }$C–20.4 ${}^{\circ }$C	26–31%
[13,14]	mobile	functional test	N/A	not available	not available
[3]	stationary	functional test	1003 hPa–1023 hPa	27.6 ${}^{\circ }$C–30 ${}^{\circ }$C	75–78%
[7]	stationary	functional test	1006 hPa–1010 hPa	31 ${}^{\circ }$C–34 ${}^{\circ }$C	54–67%
[4]	stationary	functional test	N/A	20.1 ${}^{\circ }$C–22.0 ${}^{\circ }$C	62.3–66.3%
[5]	stationary	functional test	1010 hPa–1020 hPa	−20 ${}^{\circ }$C–27 ${}^{\circ }$C	not available
[16]	mobile	not described ${}^{1}$	910 hPa–917 hPa	−2.5 ${}^{\circ }$C–−0.8 ${}^{\circ }$C	not available
[10]	mobile	functional test	N/A	25 ${}^{\circ }$C–32 ${}^{\circ }$C	32–43%
[20]	mobile	functional test	not available	15 ${}^{\circ }$C–25 ${}^{\circ }$C	not available
[8]	stationary	functional test	N/A	3 ${}^{\circ }$C–40 ${}^{\circ }$C	10–95%
[8]	mobile	functional test	not available	not available	not available
This paper	mobile	functional test	982 hPa–992 hPa	1 ${}^{\circ }$C–24 ${}^{\circ }$C	45–96%

${}^{1}$ Measurements at an altitude of about 800 m.

**Table 7:** Errors occurred during measurements carried out by primary sensors.

Day	Pressure	Temperature	Humidity	UV Index
maximum absolute error				
Day 1	0.2 hPa	0.3 ${}^{\circ }$C	1%	0.02
Day 2	0.1 hPa	0.2 ${}^{\circ }$C	2%	0.02
Day 3	0.1 hPa	0.2 ${}^{\circ }$C	1%	0.14
Day 4	0.2 hPa	0.1 ${}^{\circ }$C	1%	0.01
Day 5	0.2 hPa	0.2 ${}^{\circ }$C	1%	0.01
average relative error				
Day 1	0.02%	0.02%	0.22%	0.01%
Day 2	0.01%	0.40%	1.36%	0.13%
Day 3	0.01%	0.02%	0.13%	0.22%
Day 4	0.02%	0.55%	0.12%	0.13%
Day 5	0.02%	0.03%	0.11%	0.13%

**Table 8:** Errors reported in related work.

Paper	Station	Measurement Accuracy		
		Pressure	Temperature	Humidity
[4,6,8]	stationary	N/A	not available	not available
[8,9,20]	mobile	not available	not available	not available
[13,14]	mobile	N/A	not available	not available
[3]	stationary	1.23% ${}^{1}$	1.4% ${}^{1}$	3.85% ${}^{1}$
[7]	stationary	not available	not available	not available
[5]	stationary	not available	not available	N.A.
[16]	mobile	±5 Pa	±0.6 K	±2%
[10]	mobile	N/A	3.71%	1.65%

${}^{1}$ Maximum errors (compared with public weather station).

## Data Availability

Not applicable.

## Abbreviations

The following abbreviations are used in this manuscript:

ARM	Advanced RISC Machine;
CCC	Command and Control Console;
GCC	Google Congestion Control;
GDPR	General Data Protection Regulation;
GPIO	General Purpose Input–Output;
GPS	Global Positioning System;
HTML	Hypertext Markup Language;
HTML5	HTML version 5;
I${}^{2}$C	Inter-Integrated Circuit;
IoT	Internet of Things;
ITS	Intelligent Transportation System;
MQTT	Message Queue Telemetry Transport;
N/A	Not Applicable;
N.A.	Not Available;
NASA	National Aeronautics and Space Administration;
QoI	Quality of Information;
RISC	Reduced Instruction Set Computing;
ROS	Robot Operating System;
RTP	Real-time Transport Protocol;
SBC	Single-Board Computer;
SCTP	Stream Control Transmission Protocol;
SPA	Single-Page Application;
TFRC	TCP-friendly Rate Control;
UAV	Unmanned Aerial Vehicles;
WebRTC	Web Real-Time Communications;
WHO	World Health Organization;
WLAN	Wireless Local Area Network;
WMMS	WebRTC Multimedia and Monitoring Station;
WMO	World Meteorological Organization.
